# Outbreak of *Neisseria meningitidis* capsular group W among scouts returning from the World Scout Jamboree, Japan, 2015

**DOI:** 10.2807/1560-7917.ES.2016.21.45.30392

**Published:** 2016-11-10

**Authors:** Alison Smith-Palmer, Ken Oates, Diana Webster, Sarah Taylor, Kevin J Scott, Gemma Smith, Benjamin Parcell, Ann Lindstrand, Anders Wallensten, Hans Fredlund, Micael Widerström, Jim McMenamin, on behalf of the IMT, investigation team in Sweden

**Affiliations:** 1 Health Protection Scotland, Glasgow, United Kingdom; 2 NHS Highland, Inverness, United Kingdom; 3 NHS Grampian, Aberdeen, United Kingdom; 4 NHS Shetland, Lerwick, United Kingdom; 5 Scottish Haemophilus, Legionella, Meningococcus and Pneumococcus Reference Laboratory, Glasgow, United Kingdom; 6 International Health Regulations National Focal Point, Public Health England, London, United Kingdom; 7 Public Health Agency of Sweden, Solna, Sweden; 8 National Reference Laboratory for Pathogenic Neisseria, Örebro University, Örebro, Sweden; 9 County Council Medical Officer, Stockholm, Sweden; 10 Members are listed at the end of the article.

**Keywords:** Neisseria meningitidis, mass gatherings

## Abstract

The 23rd World Scout Jamboree was held in Japan from 28 July to 8 August 2015 and was attended by over 33,000 scouts from 162 countries. An outbreak of invasive meningococcal disease capsular group W was investigated among participants, with four confirmed cases identified in Scotland, who were all associated with one particular scout unit, and two confirmed cases in Sweden; molecular testing showed the same strain to be responsible for illness in both countries. The report describes the public health action taken to prevent further cases and the different decisions reached with respect to how wide to extend the offer of chemoprophylaxis in the two countries; in Scotland, chemoprophylaxis was offered to the unit of 40 participants to which the four cases belonged and to other close contacts of cases, while in Sweden chemoprophylaxis was offered to all those returning from the Jamboree. The report also describes the international collaboration and communication required to investigate and manage such multinational outbreaks in a timely manner.

## Introduction

Definitions of mass gatherings vary greatly, with some sources categorising any gathering of more than 1,000 individuals as a mass gathering, while others require the attendance of as many as 25,000 people to qualify. Irrespective of the definition, mass gatherings represent large numbers of people attending an event that is focused at specific sites for a finite time [1]. Mass gatherings provide the potential for disseminated outbreaks for a range of pathogens, especially respiratory and gastrointestinal [2-4]. Although meningococcal outbreaks are rarely reported from mass gatherings, there have been previous examples. An outbreak of meningococcal capsular group C was observed with 11 linked cases following a youth football tournament held in Belgium in 1997 [5]. The Hajj pilgrimages in 2000 and 2001 were associated with outbreaks of meningococcal capsular group W (MenW) [6], with a high attack rate among pilgrims and their household contacts [7]. Among those affected by the outbreak strain in England and Wales, the case fatality ratio (CFR) was 20%, significantly higher than the CFR of 9% for all other culture-confirmed cases of meningococcal disease reported in England and Wales between 1995 and 2000 [8]. In response to the outbreak in 2000, the United Kingdom (UK) Department of Health recommended MenACWY vaccine for those attending the Hajj [8].

The 23rd World Scout Jamboree was held in Yamaguchi City, Yamaguchi Prefecture, Japan from 28 July to 8 August 2015 and was attended by over 33,000 scouts from 162 countries. This included 160 scouts and 108 adults from Scotland who were either leaders or part of the international support staff. The scouts attending from Scotland comprised five distinct units, one of which was the North of Scotland unit, with 36 scouts and four adult leaders, 60% of whom were male (16 females and 24 males). The mean age of the scouts in this unit was 16.4 years (range 15–17 years).

On 12 August 2015, Health Protection Scotland was informed by a Health Protection Team in the North of Scotland of a laboratory-confirmed case of invasive meningococcal disease in a scout belonging to the North of Scotland unit who had attended the 23rd World Scout Jamboree.

### Epidemiological investigation

The epidemiological investigation carried out is illustrated in the Figure.

**Figure:**
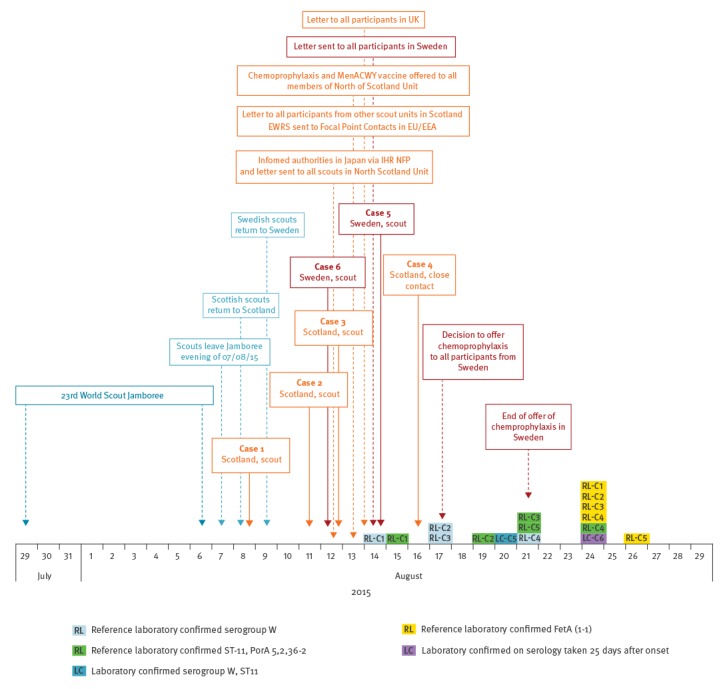
Timeline for confirmed cases of meningococcal infection among scouts returning from the World Scout Jamboree, Scotland and Sweden, 28 July to 29 August 2015 (n=6)

CSF: cerebrospinal fluid; EEA: European Economic Area; EU: European Union; EWRS: European Early Warning and Response System; IHR NFP: International Health Regulations National Focal Point; ref lab: reference laboratory; UK: United Kingdom.

### Public health action in the United Kingdom

Following the identification of the first laboratory-confirmed case (12 August), an alert was sent via email to all Health Protection Teams in Scotland to raise awareness, an information letter emailed to all scouts and leaders in the North of Scotland unit and information passed to the authorities in Japan via the UK International Health Regulations National Focal Point (IHR NFP), Public Health England. The public health response was managed according to the UK meningococcal guidance [9].

The first Incident Management Team (IMT) met on 13 August with representatives from the North of Scotland National Health Service (NHS) Boards Health Protection Teams, microbiology, infectious diseases, Scottish Government and Health Protection Scotland. Five subsequent IMTs were held with additional representation from Public Health England and Public Health Agency Northern Ireland.

Following the identification on 13 August of the second laboratory-confirmed case and the identification of four other possible cases among scouts from the North of Scotland unit a risk assessment was undertaken. The North of Scotland unit appeared to be a self-contained unit within the UK contingent travelling to and from the Jamboree. Furthermore, their tent accommodation at the site was not adjacent to the other UK scout units. Chemoprophylaxis with ciprofloxacin and MenACWY conjugate vaccine was then offered to all scouts and leaders in the North of Scotland unit in addition to other close contacts of the two confirmed cases. The decision to recommend MenACWY vaccine was based on the results of preliminary antigen detection tests from the local hospital laboratory for case one. All individuals received chemoprophylaxis, and were offered vaccination, by the end of 14 August and 18 August, respectively. Interviews conducted with these two cases did not initially identify any close contacts outside the North of Scotland unit (even for their return international flight) and the immediate households of the confirmed cases. However, a close contact of the first case in a scout unit in another area of the UK was subsequently identified on the evening of 13 August, and received chemoprophylaxis that night and MenACWY vaccine on 14 August. On the evening of 13 August an information letter was emailed to all scouts from Scotland who attended the Jamboree, providing information about the incident.

On 14 August, one of the four possible cases under investigation was confirmed to have invasive meningococcal disease and a further two possible cases identified. This brought the total to three confirmed and five possible cases. In the absence of additional information indicating joint activities with other scout groups from the Jamboree, the IMT reiterated their decision not to offer antibiotic prophylaxis to any of the other UK scouts. Following the identification of the third confirmed case, there was discussion as to whether MenACWY vaccine should be offered to all participants in the UK. A decision was made not to extend the offer of vaccination, based on no evidence of spread in the UK beyond the North of Scotland unit. Additional considerations supporting this decision included the practicalities of providing timely immunisation to such a large cohort, and concerns about vaccine availability, since a MenACWY immunisation programme had just commenced phased introduction for all 14–18 year olds and new university entrants in the UK [10]. However, using contact details provided by Scouts UK, an information letter outlining the situation and the action to take in the event of symptom development was emailed that day to the parents/guardians of all scouts, leaders and international support staff who attended the Jamboree from across the UK (ca 4,000). The rationale for this letter was that all such individuals were within the incubation period for meningococcal disease, being within 7 days of return to the UK from the Jamboree. For those individuals with no email address or for whom an undeliverable or out-of-office email response was received, alternative contact details (phone and/or postal address) were provided to the Health Protection agencies of each UK country to allow further attempts to provide information about the incident.

On 17 August, a fourth case was confirmed in a close contact of one of the scouts from the North of Scotland Unit. That scout was not a case and had reported no close contact with any of the other three confirmed cases. Chemoprophylaxis and MenACWY vaccine was provided to the close contacts of the scout’s infected close contact, including re-issuing chemoprophylaxis to the scout, due to continuing contact with the infected close contact following initial chemoprophylaxis.

Confirmed cases had a range of presenting symptoms (Figure). It was observed that a number of cases had respiratory symptoms such as cough or sore throat. As a result it was decided that there should be a low threshold for treating possible cases who presented with respiratory symptoms.

None of the remaining five possible cases under investigation were confirmed as invasive meningococcal disease or, on further review, clinically considered to be a case of invasive meningococcal disease; one had a positive throat swab for group G streptococci, one a positive throat swab for rhinovirus/enterovirus (combined test does not determine which is positive). All four confirmed cases made a rapid clinical recovery after admission to hospital and were discharged from hospital by 20 August.

A total of 53 individuals in Scotland and one outside Scotland received chemoprophylaxis and were offered vaccination.

### Microbiological investigations in Scotland

*Neisseria meningitidis* isolates submitted to the Scottish *Haemophilus*, *Legionella*, Meningococcus and Pneumococcus Reference Laboratory (SHLMPRL) by regional diagnostic microbiology laboratories were characterised by standard phenotypic procedures (api NH (BioMérieux), Wellcogen *N. meningitidis* ACYW135 latex reagent (Remel Europe Ltd.) and monovalent meningococcus agglutinating serum (Remel Europe Ltd.)). Confirmation of *N. meningitidis* isolates and detection of *N. meningitidis* DNA in clinical specimens was determined by *ctrA* PCR [11]. Genotypic capsular grouping was performed by *siaD* PCR [12] *siaD_W135_* and *siaD_Y_* primer and probe information was kindly provided by Dr Malcolm Guiver at the PHE Meningococcal Reference Unit (Manchester, UK). Multilocus sequence typing (MLST), PorA variable region (VR) sequencing and FetA VR sequencing were performed as outlined on the *Neisseria* sequence-typing website [13].

All four isolates from the confirmed cases were indistinguishable by the phenotypic and molecular typing procedures outlined above. Based upon the EMGM-recommended strain designation [14] this identified the *N. meningitidis* strain as W: P1.5,2,36-2: F1-1: ST-11 (cc11).

Preliminary typing suggested that the W strain is indistinguishable from that responsible for the recent increase in MenW ST-11 disease in England and Wales since 2009 and more recent indications of increased disease in Scotland, with 15 isolates of MenW ST-11 reported in Scotland in the first 45 weeks of 2015, accounting for 24% of all isolates over the time period, compared with just five, accounting for 7% of cases in 2014 (SHLMPRL, data not shown) 

### International aspects

On the evening of 13 August, a European Early Warning and Response System (EWRS) message was circulated to the National Focal Point contacts in the European Union (EU)/European Economic Area (EEA) about the meningococcal cases in Scotland.

#### Public health action in Sweden

On 14 August the Public Health Agency of Sweden, with the help of the Swedish Scout Organisation, distributed a letter to the returning scouts recommending them to seek healthcare promptly upon signs of meningitis illness. On Sunday 16 August, the first Swedish case (case 5, Figure), a scout who had attended the Jamboree, was admitted to hospital with a clinical picture of meningitis and shock, with date of onset of first symptoms (not including shock) of 14 August. The case was treated in intensive care for 6 days. The case finally recovered well and was discharged on 28 August. Gram-negative diplococci were initially found in CSF and *N. meningitidis* was verified by PCR the next day and subsequently by culture. The isolated strain was confirmed as capsular group W with the PorA profile 5,2,36-2, the same as identified from the four Scottish cases. This strain had also been previously isolated in Sweden in 2014 and 2015.

On the evening of 16 August the Public Health authorities received reports of a second suspected case, a scout leader who became unwell on 13 August and was hospitalised on 15 August due to suspected septicaemia. The cases and their close contacts were managed according to the national guidance [15,16].

On the morning of 17 August, an urgent teleconference was convened by members of Communicable Disease Control and Prevention in Stockholm and Gothenburg, the Public Health Agency of Sweden and the National reference laboratory for Pathogenic *Neisseria* in Örebro, Sweden. During the one-hour meeting, two more suspected cases of meningococcal septicaemia were reported; one scout hospitalised in the South of Sweden and one scout hospitalised in Stockholm. This latter case was positive for *N. meningitidis* capsular group W with PorA profile 5,2,36-2 in throat swab (result available on 26 August) and later confirmed by serology (case 6, Figure). Thus at that point in time the authorities in Sweden were aware of one confirmed and three suspected cases that appeared to be from different units and all hospitalised within 24 hours. The authorities were therefore unable to define a limited high-risk group among the scouts. Further, the cases had occurred within a high-risk setting for transmission with young people living in camp conditions and close social interaction. Hence a decision was taken to recommend ciprofloxacin prophylaxis to all 1,900 scouts across Sweden. It was also decided when possible to obtain a throat swab to find out the carriage rate in such an outbreak, as this had not had been investigated in Sweden in modern times and there was now a unique opportunity to find out the carrier state of meningococci in teenagers in Sweden, a low-incidence country for invasive meningococcal disease (0.5/100,000 inhabitants in 2014). These screening results will be published at a later date.

The offer of free prophylactic antibiotics in Sweden ended on 21 August, after which the risk of further cases due to transmission in Japan was deemed to be very unlikely. On the same day a questionnaire was administered to all participants in order to determine how many had taken up the offer, and how many had a throat swab taken, in addition to assessing general satisfaction with information and service delivery. Data from Sweden indicate that chemoprophylaxis uptake was around 80%, and that more than 90% were satisfied with the information and instructions provided by the authorities. However, there were reports from healthcare providers in Sweden that the information about the intervention had not been received in all clinics.

Follow-up of the two confirmed Swedish cases identified that they belonged to the same scout unit. This information had not been available when the decision to offer chemoprophylaxis was made.

The first confirmed Swedish case was later identified as having attended a cultural day at the campsite on 2 August. The cultural exchange day comprised an interfaith ceremony and a food festival in the afternoon during which scouts cooked their own traditional dishes and invited scouts from other countries to taste and experience food and cultural differences among countries. Scouts from all countries were asked to walk around the sub-camp to mingle and taste food from different countries, and during this event they visited the North of Scotland unit and tried their food and drink.

The organisers also held discotheques every third evening during the Jamboree. Anecdotal evidence also suggested extensive mixing between participants from many countries in keeping with the international nature of the meeting.

Examination of the campsite plan revealed that the North of Scotland unit had slept in tents in the western hub of the camp, as had the two confirmed cases from Sweden, although they were not immediately adjacent. The units closest to the North of Scotland unit were from the United States, Hong Kong, Japan, France, Luxembourg and Pakistan, none of which reported any cases

The two possible cases from Sweden were negative for *N. meningitidis* and subsequently discounted by the authorities as meningococcal disease.

#### Wider international aspects

Throughout the investigation, regular updates were issued via EWRS to EU/EEA countries, and information exchanged, through the IHR NFP, with authorities in Japan. The EWRS alerts provided a rapid mechanism for both disseminating and collating information. In response to the EWRS, 20 countries reported that they had issued information to participants to raise awareness of the signs and symptoms. None of these 20 countries recommended antibiotics and no associated meningococcal cases were reported.

The Ministry of Health, Labour and Welfare in Japan requested that the Scout Association of Japan alert participants to be aware of the signs and symptoms of meningococcal disease and liaised with the Jamboree organisers. The Jamboree organiser provided information to units who had stayed near the North of Scotland unit. There were no cases of invasive meningococcal disease reported in Japan associated with the Jamboree.

## Discussion

The North of Scotland scouts were not vaccinated against MenW before the Jamboree, since the UK recommendations on immunisation of travellers do not include Men ACWY vaccination in these circumstances. Likewise, neither of the Swedish cases was vaccinated. However, although vaccination is not recommended in Sweden before mass gathering events, a small number of Swedish scouts had been vaccinated before the trip. 

In response to a recent UK increase in MenW disease, in February 2015 the UK advisory body on immunisation the Joint Committee on Vaccination and Immunisation recommended a vaccination programme aimed at protecting adolescents against meningococcal capsular groups ACW and Y strains. This was felt to be the best option to generate population-level protection since teenagers are in the age group with highest meningococcal carriage levels, and also an age group at increased risk of disease [17]. This recommendation was accepted by the UK Departments of Health. The immunisation programme in Scotland for 14–18-year-olds started in August 2015 for young people who had left school, whether attending full-time education or not, and others aged < 25 years starting university for the first time, and the school-based programme began in January 2016 [10] This programme has replaced the earlier MenC immunisation offered as an adolescent booster in schools with a catch-up programme. Therefore, in future years, adolescents from the UK attending Jamborees and similar mass-gathering events should be protected against these capsular groups. As similar programmes are not currently in place in all other countries it will be for individual countries to consider whether there should be local recommendations for those attending such events.

The rapid communication of the identification of meningococcal disease among participants of a dispersed mass gathering allowed public health authorities to target information to the international Scout Movement attendees of the Jamboree in individual countries. This timely dissemination led to rapid identification of other Scottish cases, and facilitated the identification of an epidemiological link to the Swedish case. In both the UK and Sweden the excellent electronic records and cooperation of scouting organisations greatly facilitated this process and allowed the rapid dissemination of information to participants. However it is recognised that for many mass gatherings where similar outbreaks may occur, for example music festivals, sports events and religious celebrations, such comprehensive contact lists will not be available, making it extremely difficult to identify and contact potentially exposed individuals within the critical incubation window.

It was of interest that the IMT in Scotland and the rest of the UK arrived at different decisions than Sweden in terms of the extent of chemoprophylaxis offered. In Scotland the risk assessment for the cases, which were restricted to the North of Scotland, limited this offer to a small group, whereas in Sweden all 1,900 Jamboree attendees were offered chemoprophylaxis as it was not possible to identify a specific cohort at increased risk, as the information available on 17 August suggested four cases under investigation hospitalised in the previous 24 hours from different units.

Uptake of chemoprophylaxis was high in both Scotland and Sweden and administered in a timely manner. Unfortunately it is not possible to determine if the mass distribution of prophylaxis prevented further cases. Comments from Sweden that not all healthcare providers had received the appropriate information highlight the importance of clear communication channels between public health institutions and healthcare systems, and the practical issues of conducting large exercises with tight timescales

In a previous analysis of 129 UK MenW cases, none were contacts of another MenW case [18], making this the largest cluster (n = 6 confirmed cases; 4 in Scotland) in this current UK increase in MenW disease. Most individuals infected with *N. meningitidis* experience a period of asymptomatic carriage with no disease. A meta-analysis of carriage prevalence has shown increased carriage throughout childhood from 4.5% in infants to a peak of 23.7% in 19 year-olds subsequently decreasing in adulthood to 7.8% in 50 year-olds [19], similar levels for 19–25 year-olds of 26.5% were reported from a UK carriage study in 2011, with capsular groups B and Y the most common at that time [20]. Although meningococcal carriage is potentially high in the participant age group, with carriage also depending on exposure to smoking, intimate kissing, pub-/club-type social settings and coincident respiratory tract infections of viral or bacterial origin [21], it is unclear why these cases developed invasive disease. Extensive social mixing associated with the Jamboree, preceding viral/bacterial infection and long-haul air travel could have been contributing factors.

Evidence from the UK increase of this sequence type (ST11), has suggested an often atypical clinical presentation, with initially mild symptoms for some cases, and a case fatality rate of 12% [18], lower than that previously reported from the MenW outbreak associated with the Hajj [8]. The four confirmed cases from Scotland tended to have an atypical presentation, dominated by respiratory symptoms and did not have a severe course of disease, with none requiring intensive care admission. It is possible the latter may reflect early clinical presentation in response to the public health alert and prompt antibiotic intervention. Interestingly, in the previous analysis of UK MenW cases, such respiratory presentations were also associated with less severe disease [18]. Whole-genome sequencing is underway to further characterise the outbreak isolates from Scotland and allow more detailed comparison with the endemic UK strain. These data may help explain apparent associations between clinical presentation, severity and outcome. Continued enhanced surveillance in this area will be important.

### Public health recommendation: decisions on the need for mass prophylaxis and vaccination

Decisions on the need for mass prophylaxis in large events like this need to be taken rapidly, even if only limited information is available initially. Each situation is likely to be different and will not be predictable. While UK guidance addresses such contingency, further work on development of generic decision algorithms should be considered. If a decision is taken to recommend prophylaxis or vaccination it is important that information can be delivered quickly, within hours to both those who may be at risk, and also to the healthcare system so they can arrange for delivery of the service in a timely manner.

## Notes

**Members of the Scotland Incident Management Team and investigation team in Sweden:** Claire Cameron, Health Protection Scotland. Syed Ahmed, Health Protection Scotland. Heather Murdoch, Health Protection Scotland. John Schofield, Health Protection Scotland. Duncan McCormick, Scottish Government. Lynsey MacDonald, Scottish Government. Diane Lindsay, Scottish *Haemophilus, Legionella*, Meningococcus and Pneumococcus Reference Laboratory. Andrew Smith, Scottish *Haemophilus, Legionella*, Meningococcus and Pneumococcus Reference Laboratory. Roisin Ure, Scottish *Haemophilus, Legionella,* Meningococcus and Pneumococcus Reference Laboratory. Abhayadevi Tissington, NHS Highland. Jenny Wares, NHS Highland. Chin Lim, NHS Highland. Lucy Denvir, NHS Tayside. Margaret Ramsey, NHS Tayside. Isabell MacInnes, NHS Western Isles. Ivan Tonna, NHS Grampian. Jillian Johnston, Public Health Agency, Northern Ireland. Neil Irvine, Public Health Agency, Northern Ireland. Sema Mandal, Public Health England. Gemma Smith, Public Health England. Shamez Ladhani, Public Health England. Mary Ramsay, Public Health England. Per Follin, County Council Medical Officer, Gothenburg. Cecilia Jernberg, Public Health Agency of Sweden. Anna-Lena Hammarin, Public Health Agency of Sweden. Susanne Jacobsson, National Reference Laboratory for Pathogenic Neisseria, Örebro University, Sweden.
